# Therapeutic Manipulation of the Atrial Septum: Tearing Down the Wall

**DOI:** 10.1016/j.jscai.2026.105335

**Published:** 2026-04-07

**Authors:** Cole Buchanan, Ryan J. Tedford, Sheldon E. Litwin

**Affiliations:** a Division of Cardiology, Medical University of South Carolina, Charleston, South Carolina; b Division of Cardiology, Ralph H. Johnson Veterans Affairs Health System, Charleston, South Carolina

**Keywords:** atrial shunting, congestive heart failure, pulmonary arterial hypertension

A septum is defined as a dividing wall or membrane especially between bodily spaces or masses of soft tissue. In the heart, the atrial septum is a thin muscular and membranous structure dividing the left and right atria. The septum is composed of the septum primum and septum secundum. During fetal life, the foramen ovale allows passage of oxygenated blood from the right atrium to the left atrium to bypass the fetal lungs. After birth, increased left atrial pressure causes closure of the foramen ovale, and ultimately there is fusion of the septum primum and septum secundum in 75% to 85% of people. The fossa ovalis, the adult remnant of the foramen ovale, is a roughly oval-shaped portion of the septum ∼1.5 cm in diameter. It is the thinnest portion of the atrial septum and is frequently used by cardiologists to create a temporary or permanent passage between the left and right atria.

In the 1960s,Rashkind et al^1^first described the use of balloon atrial septostomy to allow mixing of oxygenated and deoxygenated blood in infants with congenital heart defects, such as D-transposition of the great arteries or other cyanotic defects. This lifesaving procedure allows oxygenated blood to be delivered to the body in babies with separated venous and arterial circulations. It has been said that the medical community’s initial response to Rashkind’s report “…varied between admiration and horror…”^2^ This work is generally considered to be the origin of interventional cardiology. In current practice, balloon atrial septostomy is occasionally performed in patients with refractory pulmonary arterial hypertension as a bridge to transplant or palliation.

Although the Rashkind procedure is used primarily to provide mixing of oxygenated and deoxygenated blood, other related approaches utilizing atrial septostomies have been developed to serve as pressure release valves for overloaded portions of the heart. The Fontan procedure, first developed in 1968, is used to divert inferior vena cava and superior vena cava blood flow directly to the pulmonary artery, bypassing the heart in children with functional single ventricles. Pressures within the Fontan conduit can be very high, leading to postoperative complications such as high amounts of chest tube drainage and long-term complications such as pleural effusions and protein-losing enteropathy. Creation of a 3- to 6-mm hole or fenestration within the Fontan shunt has been used to produce a pop-off valve that lowers the Fontan pressures and may increase cardiac output by augmenting preload to the systemic ventricle and limiting excessive systemic venous pressure. Although the creation of a fenestration has demonstrated benefits, there are also risks, and the presence of right-to-left shunting may cause complications such as hypoxia or paradoxical embolism.

In the modern era, transseptal puncture is an increasingly used technology that allows performance of common transcatheter procedures such as atrial fibrillation ablation, left atrial appendage occlusion, and mitral valve edge-to-edge repair.^3^ Most often 8F to 12F chatheter (2.6 to 4.0 mm diameter) sizes are used for transseptal procedures; however, some procedures use up to 22 F catheters (larger). With smaller punctures, the residual defects close within months in majority of patients; however, larger catheter sizes may produce hemodynamically significant defects.

Over the past decade, there has been a rising interest in the potential to use therapeutic atrial shunting to decompress the pressure-loaded left atrium in patients with heart failure.^4^^,^^5^ The hypothesis has been that elevated left atrial pressure, particularly during exercise, is a fundamental cause of exertional dyspnea, the cardinal symptom of heart failure. It has been proposed that in carefully selected patients, creation of a small atrial septal defect (5 to 10 mm) will allow shunting of blood from the left to right atrium, thus lowering left atrial and pulmonary vascular pressures. Because the right heart is generally thinner and more compliant than the left heart, transiently increasing the volume in the right heart may be better tolerated than elevations in left atrial pressure. Theoretically, an atrial shunt can act as both a sensor of high left atrial pressure and a means to lower that pressure in real time. This is very attractive to clinicians and patients. In the ideal patient, in whom left atrial pressure is relatively low and close to right atrial pressure under resting conditions, little shunting will occur during periods of inactivity. However, when the patient begins exercising and left atrial pressure abruptly increases, the shunt can allow immediate left atrial decompression. It is hoped that appropriate patient selection can minimize the long-term consequences of left-to-right shunting while producing symptomatic benefit.

The REDUCE LAP HF II trial randomized patients with heart failure and ejection fraction >40% to atrial shunt or a sham procedure.^4^^,^^6^^,^^7^ Although the overall trial was neutral, a “responder” group was identified.^8^ A key characteristic of the responder group was a low pulmonary vascular resistance during exercise (<1.74 WU). Patients with high exercise pulmonary vascular resistance had worse outcomes with shunting. These data were interpreted as showing that a compliant pulmonary vasculature and right heart were necessary to accommodate the left-to-right shunting. An interesting observation seen after the creation of a left-to-right shunt was an unexpected improvement in pulmonary vascular compliance despite the increased blood flow through the pulmonary circulation.^9^ This finding has been attributed to the delivery of more highly oxygenated blood into the pulmonary vasculature, which then relieves any hypoxic pulmonary vasoconstriction that might result from reduced mixed venous oxygen saturation that is often seen in heart failure.

In the current issue of *JSCAI*, Bartunek et al^10^ report the use of an adjustable atrial shunt device (Occlutech Atrial Flow Regulator, Occlutech Holding AG) in 24 patients with advanced pulmonary arterial hypertension, maximally tolerated medical therapy, poor prognosis, and limited additional therapeutic options. The goal in this population was to produce right-to-left shunting, thereby decompressing the overloaded right heart. Although the direction of the shunt is opposite, the fundamental tactic of decongesting the overloaded side of the heart is similar to the approach that is being studied in patients with predominant left heart failure. Three patients died from complications related to the procedure. Nonetheless, in this very sick group of patients, the creation of an adjustable 6- to 8-mm atrial shunt was associated with remarkable improvement in hemodynamics and functional status in those who survived after the creation of the shunt. At 3 months, atrial flow regulator implantation reduced pulmonary vascular resistance and improved cardiac index despite lower arterial oxygen saturation. Echocardiography showed smaller right ventricular diameter, lower right ventricle (RV)/ left ventricle ratio, higher tricuspid annular plane systolic excursion and RV fractional area change, and improved RV–pulmonary artery coupling. N-terminal-pro brain natriuretic peptide levels decreased, NYHA class improved (>1 class) in 66% of patients, and 6-minute walk distance increased. During a 1-year follow-up, 9 patients died and 3 underwent lung transplantation. The results of the study are thought provoking because patients with heart failure with preserved ejection fraction (HFpEF) and high pulmonary vascular resistance, the hallmark of pulmonary arterial hypertension, seemed to fare poorly in the REDUCE LAP HF 2 study.^8^

How do we interpret these interesting data and put them into context with the results of the HFpEF trials? One possibility is that there is a U-shaped relationship between pulmonary vascular resistance and the benefits attributable to the creation of an atrial level shunt with net benefit to patients on either end of the spectrum with predominant left or right-sided disease (Figure 1). Patients with predominant left heart failure and low pulmonary vascular resistance may reap the benefits of intermittent left-to-right shunting due to left atrial decompression. In contrast, in patients with mixed or intermediate levels of left heart and right heart disease and moderately elevated pulmonary vascular resistance, there may be little benefit to producing a shunt because the right heart and pulmonary vasculature cannot accommodate the additional blood flow. At the other end of the spectrum, patients with advanced pulmonary vascular and right heart disease may benefit from decompressing the markedly overloaded right heart and potentially improving preload to the underfilled left ventricle. Based on the impressive results of the current study, the hemodynamic improvements appear to outweigh the mild degree of arterial hypoxemia, at least in the short term.

**Figure 1:**
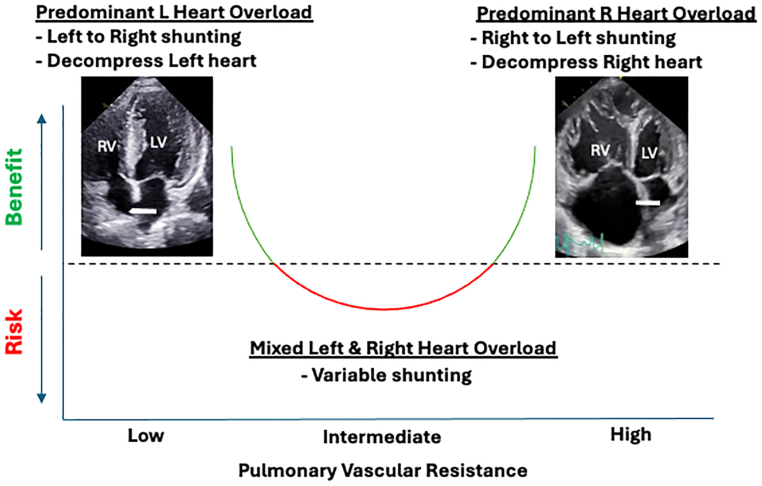
**Hypothetical relationship between degree of left and right heart overload and risks and benefits of interatrial shunting.** Based on observations from this and other published trials, it is proposed that patients with relatively pure left or right heart overload may benefit from shunting in opposite directions, whereas those with intermediate degrees of left and right heart disease may not benefit or may even be harmed by shunting. LV, left ventricle; RV, right ventricle.

Another interesting aspect of the current study is the use of an adjustable device in which the size of the shunt orifice can be manipulated after implantation.^10^ This unique feature of the shunt device used in this study addresses one of the key challenges in the field of therapeutic atrial shunting. Computer simulations suggested an 8-mm orifice would produce optimal but controlled amounts of left-to-right atrial shunting in HFpEF^11^; however, there are not enough human data with different-sized shunts to determine whether shunt size needs to be individualized based on patient characteristics, such as body size, heart size, cardiac output, and intracardiac pressures. The ability to adjust the shunt orifice after implantation is theoretically very appealing because there are frequent undulations in hemodynamics in patients with heart failure or pulmonary hypertension. Moreover, a risk of balloon atrial septostomy in pulmonary arterial hypertension is causing too much shunting and significant hypoxemia, so an adjustable shunt that is theoretically closable could add a margin of safety to this approach. Unfortunately, the small size of this trial and vaguely defined criteria guiding the choice of shunt diameter do not provide new insights into ways to personalize the choice of shunt sizes.

In conclusion, there has been rapid growth in the use of the atrial septum for both procedural access to the left heart and for investigational therapeutic purposes. Creation of permanent atrial shunts may be used to decompress and decongest overloaded left- or right-sided cardiac chambers, with the direction of shunting depending on the predominant pathology. Much work remains to be done to identify, with certainty, which, if any, patients benefit most from these types of procedures. Careful study will also be required to optimize shunt size and location so that maximal gain and minimal harm will result from these novel approaches to the treatment of heart conditions that previously had limited therapeutic options.

## Declaration of competing interest

Cole Buchanan has no relationships to disclose. Ryan Tedford has consulting relationships with and receives honorarium/consulting fees from Abbott, Acorai, Adona, Aria CV Inc, Boston Scientific, CVRx, Endotronix, Edwards Lifesciences, Fauna Bio, Gradient, Imbria, Medtronic, Merck, Morphic Therapeutics, Pulmovant, Restore Medical, Tempus AI, 35Pharma, and United Therapeutics. He serves on steering committees for Abbott, Edwards, Endotronix, Gradient, Merck, Restore Medical, and Tempus AI as well as a research advisory board for Abiomed. Sheldon Litwin has consulting relationships with Alleviant, Axon, Corvia Medical, Intershunt, Novo Nordisk, and Lunair.
